# A Semi-Automatic Labeling Framework for PCB Defects via Deep Embeddings and Density-Aware Clustering

**DOI:** 10.3390/s25206470

**Published:** 2025-10-19

**Authors:** Sang-Jeong Lee, Sung-Bal Seo, You-Suk Bae

**Affiliations:** 1 Multimodal AX Business Team, LG CNS Co., Ltd., Seoul 07795, Republic of Korea; sjlee89@lgcns.com; 2 Department of Computer Engineering, Tech University of Korea, Siheung 15073, Republic of Korea; sungbal@tukorea.ac.kr

**Keywords:** printed circuit board inspection, semi-automatic labeling, clustering, ResNet-50, vision transformer, class imbalance

## Abstract

**Highlights:**

**What are the main findings?**

- A production-oriented, semi-automatic labeling pipeline reliably converts defect ROIs into consistent class labels by coupling margin-aware cropping, pretrained embeddings, and clustering, achieving cluster-level label quality without dense, pixel-wise annotation.

**What is the implication of the main finding?**

- Cluster-level decisions concentrate human effort where it matters—on ambiguous, low-consistency clusters—thereby reducing labeling latency while maintaining label fidelity.

**Abstract:**

(1) Background. Printed circuit board (PCB) inspection is increasingly constrained by the cost and latency of reliable labels, owing to tiny/low-contrast defects embedded in complex backgrounds and severe class imbalance. (2) Methods. We proposed a semi-automatic labeling pipeline that converts anomaly detection proposals into class labels via small margin cropping from images, interchangeable embeddings (HOG, ResNet-50, ViT-B/16), clustering (k-means/GMM/HDBSCAN), and cluster-level verification using representative montages. (3) Results. On 9354 cropped defects spanning 10 categories (imbalance IR ≈ 1542, Gini ≈ 0.642), ResNet-50 + HDBSCAN achieved NMI ≈ 0.290, AMI ≈ 0.283, and purity ≈ 0.624 with ~47 clusters; ViT + HDBSCAN was comparable (NMI ≈ 0.281, AMI ≈ 0.274, ~44 clusters). With a fixed taxonomy, k-means (K = 10) yielded the strongest ARI (0.169 with ResNet-50; 0.158 with ViT). Macro-purity exceeded micro-purity, indicating many small, homogeneous clusters suitable for one-shot acceptance/rejection, enabling an upper-bound ~200× reduction in operator decisions relative to per-image labeling. (4) Conclusions. The workflow provides an auditable, resource-flexible path from normal-only localization to scalable supervision, prioritizing labeling productivity over detector state-of-the-art and directly addressing the industrial bottleneck in the development lifecycle for PCB inspection.

## 1. Introduction

Visual inspection (VI) of printed circuit boards (PCBs) is critical to modern electronics manufacturing because even minute defects can propagate into costly field failures. Building robust vision models for VI is, however, label-intensive: operators must curate fine-grained labels at scale under severe class imbalance and rapidly evolving product variants. Two imaging factors amplify this burden. First, PCB images present highly intricate copper-trace backgrounds, which camouflage defect boundaries and generate challenging negatives. Second, many defects are tiny and low-contrast relative to the board area, making instance-level annotation slow and error-prone, while naive cropping may discard crucial context. In production, the practical bottleneck is, therefore, less about model capacity than about the cost and latency of reliable labels [1,2,3].

Defect localization in industrial imagery has been extensively investigated within the small object detection paradigm, wherein models are trained exclusively on tiny defect-included data [4,5,6]. One-class and reconstruction paradigms and patch-distribution modeling—representatively deep one-class objectives, PaDiM, PatchCore, and DRAEM—have demonstrated strong localization capability without using defect labels [7,8,9,10]. Yet, these methods mostly terminate at localization; they seldom specify procedure-level mechanisms for converting proposals into consistent class labels suitable for training downstream detectors, auditing data quality, or supporting iterative taxonomy changes in production.

A complementary line of work employs semi- or weakly-supervised methods to reduce annotation effort by coupling pretrained embedding extraction with clustering, thereby enabling labeling at the cluster rather than the image level. Embeddings extracted from pretrained backbones (e.g., ResNet-50 [11] or ViT-B/16 [12]) are grouped by partitioning or density methods, enabling cluster-level rather than image-level labeling. Although this strategy reduces labeling effort in principle, operation constraints often limit deployment. (i) Few works offer automatic cluster–granularity selection (e.g., auto-K or robust density heuristics), (ii) memory/latency issues appear at the scale of thousands of images, and (iii) human-centered visualization for rapid verification (e.g., representative montages) is typically absent.

In our previous study [13], we developed the first half of this workflow: an anomaly detection stage trained solely on defect-free PCB board (“OK”) images and an inference stage that proposes defect regions of interest (ROIs) on production imagery, thereby reducing the search space for human reviewers without using defect labels. A critical gap remained—turning proposals into consistent, class-level supervision suitable for training and validating downstream detectors. The present paper proposes the second half of the pipeline, converting defect ROIs into labels through a semi-automatic, cluster-centric process customized to PCB imagery. Our objective is not to achieve state-of-the-art detector accuracy but to maximize labeling productivity while preserving label quality sufficient for downstream training.

The main contributions of the present study are summarized as follows:

(i) An end-to-end, production-oriented workflow that connects ROI proposals to batch labels via margin-aware cropping, backbone-agnostic embeddings (Histogram of Oriented Gradients (HOG) [14,15]/ResNet-50/ViT-B/16), auto-configurable clustering (k-means [16,17]/Gaussian Mixture Model (GMM) [18,19]/Hierarchical Density-Based Spatial Clustering of Applications with Noise (HDBSCAN) [20,21]), and cluster-level visualization (top-K montages) for rapid human confirmation was used.
(ii) Operational mechanisms include data-scale heuristics for HDBSCAN and memory-safe incremental PCA for HOG.
(iii) A comprehensive evaluation on a real PCB corpus with 9354 crops across 10 categories (long-tailed distribution) is performed, reporting agreement metrics alongside t-SNE projections and montage-based qualitative analyses.

### Related Works

Beyond semi-automatic labeling via label propagation-based semi-supervised learning (SSL) and active learning (AL), modern self-supervised encoders are directly relevant because our pipeline relies on transferable embeddings. MoCo (momentum dictionary with queue) [22], SimCLR (augmentation-driven contrastive learning without memory bank) [23], and DINO (self-distillation for vision transformers) [24] are widely adopted foundations that improve downstream defect recognition from limited labels. In addition, PCB-specific public datasets underpin reproducible evaluation. DeepPCB (1500 template–test pairs; six defect types) [25] and HRIPCB/PCB defect-style sets (~1.3k images; six canonical classes) [26,27] are commonly used benchmarks in the literature.

Semi-automatic labeling for PCB inspection is typically instantiated as (i) semi-supervised learning (SSL) with label propagation/LGC and (ii) active learning that queries the most informative samples for expert review. Classic label propagation and LGC [28,29] and a deep label propagation approach [30] are standard SSL baselines, and a PCB-specific SSL study shows that mixing unlabeled boards reduces labeling effort while maintaining accuracy [31].

Our previous study [13] (published in The Transactions of the Korean Institute of Electrical Engineers, 2024, in Korean) focused on developing an anomaly detection-based visual inspection pipeline for PCB manufacturing. That work primarily compared backbone architectures (ResNet50 with CBAM and SpinalNet variants) and two classical anomaly detection models (PaDiM and PatchCore) using supervised and unsupervised evaluation metrics. The main contributions were (i) identifying an optimal feature extractor for PCB texture representation and (ii) proposing a mean-shifted contrastive loss function that improved anomaly detection accuracy under limited defect samples. However, the earlier framework still required pixel-level or image-level labels for evaluation and did not address labeling cost or cluster consistency.

## 2. Materials and Methods

We began from anomaly localization outputs produced by the earlier stage [13], which was trained only on normal images and yielded candidate bounding boxes on production boards. This study focused on converting those proposals into scalable supervision via cropping, embedding, clustering, and human verification.

### 2.1. Pipeline Overview

**Figure 1:**
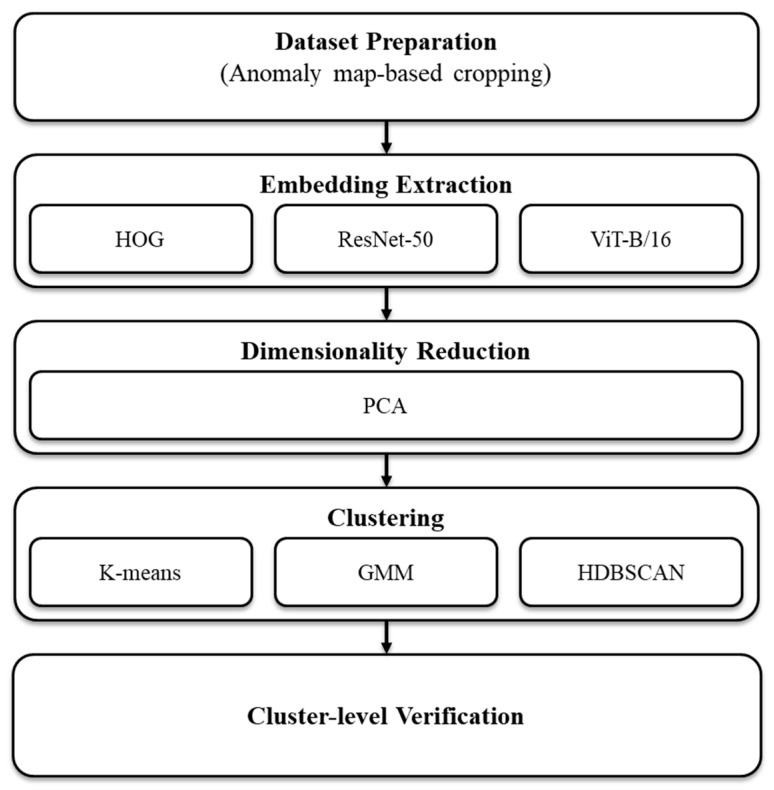
Overview of the semi-automatic labeling workflow.

Figure 1 illustrates the overall semi-automatic labeling pipeline from cropping and embedding to clustering and cluster-level verification (Appendix A). Each detected box was expanded by 10 pixels and cropped from the image to preserve minimal context around tiny defects while limiting background noise. Crops were resized to 224 × 224, normalized per backbone, and embedded. The resulting vectors were clustered; assignments, per-cluster folders, and top K-representative montages (closest to the centroid for partitioning methods or to the medoid for HDBSCAN) were exported, enabling annotators to label by cluster rather than by image, substantially reducing the number of clicks and review time.

### 2.2. Dataset Preparation

The dataset consisted of 9354 cropped patches derived from defect ROIs by expanding the proposal bounding boxes by 10 pixels on all sides prior to extraction.

**Figure 2:**
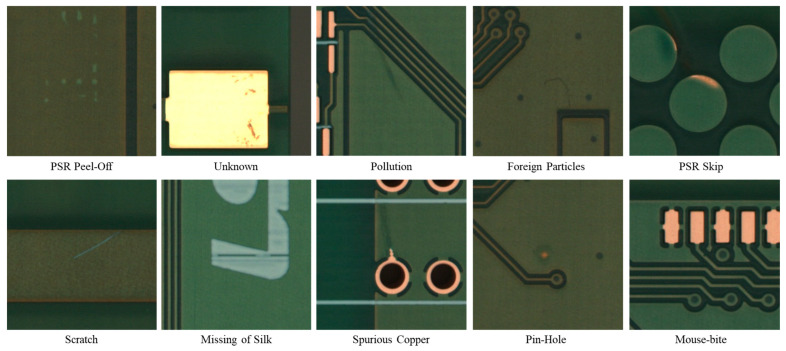
Representative examples for each defect category.

As shown in Table 1, the distribution was markedly long-tailed: PSR Peel-Off, Unknown, Pollution, Foreign Particles, PSR Skip, Scratch, Missing of Silk, Spurious Copper, Pin-Hole, and Mouse-bite—ten categories in total. Representative examples of each defect category are shown in Figure 2.

We quantified dataset imbalance using two complementary statistics. First, the imbalance ratio [32] was defined as IR = n_max/n_min, i.e., the ratio between the majority and minority class sizes [33]. Second, we report the Gini coefficient of the class-count distribution as follows:

(1) $$G=\;\frac{\sum_{i=1}^{K}\sum_{j=1}^{K}\left|n_{i}-n_{j}\right|}{2K\bar{n}}$$

where K is the number of classes and is the mean class count; this standard inequality measure summarizes dispersion beyond the extremes.

All images were initially labeled by Annotator-1 according to the defect taxonomy. To assess labeling reliability, a subset of 2269 patches was independently annotated by a second rater (Annotator-2) under the same protocol. Inter-rater agreement was quantified using Cohen’s κ [34].

### 2.3. Embedding Extraction

We evaluated three interchangeable backbones. HOG provides an efficient, hardware-agnostic baseline. We used standard cell/block/bin settings and applied incremental PCA to reduce dimensionality (e.g., 128–256) with optional float [35] storage for memory saving [25]. ResNet-50 yielded 2048-D global average-pooled features from an ImageNet-pretrained network, providing a favorable trade-off between separability and computational cost [11]. We removed the classification head of ViT-B/16 and served it as a generic encoder [12]. All backbones shared the same resizing and normalization protocol, and fixed seeds were used where stochasticity is present (Table 2).

### 2.4. Clustering Algorithms and Implementation

We adopted complementary methods matched to different regimes (Table 3). K-means minimizes within-cluster squared distances and is simple to implement when class cardinality, which denotes the number of samples per class. We accordingly set K via defect labels and fix seeds for stability [16,17]. GMM offered a probabilistic alternative better suited to anisotropic structures to keep memory bounded on high-dimensional embeddings [18,19]. We employed diagonal covariance and the same automatic cluster numbering mechanism. As a density-based method that labels noise rather than forcing assignments, HDBSCAN often yields numerous small, high-purity clusters, which is advantageous for batch labeling under extreme class imbalance [20,21].

### 2.5. Accuracy Evaluation

Agreement with the reference was assessed using Purity, as well as NMI/AMI [36] and ARI [37]. For topology inspection, we computed t-SNE [38] embeddings with fixed random seeds and a reasonable perplexity, supplemented, where appropriate, by an additional dimension-reduction method. Qualitative analysis relied on top 10 cluster montages that represented intra-cluster consistency and frequent failure modes (e.g., mixing between visually similar classes, background-driven grouping, and over-fragmentation of minority classes).

In addition, we designed controlled imbalance scenarios to assess the robustness of the proposed clustering pipeline. Specifically, we constructed down-sampled subsets with IR = 500 and IR = 1000 by randomly reducing the majority classes while preserving all minority classes. This process maintained class diversity while modulating imbalance severity, enabling a systematic analysis of how cluster purity and information-theoretic metrics vary across imbalance levels.

## 3. Results

### 3.1. Comparative Analysis of Embedding Backbones and Clustering Algorithms

Table 4 demonstrates that ResNet-50 paired with HDBSCAN showed the highest information-theoretic criteria among the tested configurations (e.g., NMI ≈ 0.290, AMI ≈ 0.283) with a relatively large number of discovered clusters (~47), consistent with HDBSCAN’s preference for small, high-purity groups. When the class cardinality was known, k-means with K = 10 showed the strongest ARI (e.g., 0.169 with ResNet-50; 0.158 with ViT-B/16), reflecting superior label-wise partition agreement under a fixed K. ViT-B/16 tracked closely behind ResNet-50 across both HDBSCAN and k-means. In contrast, the HOG baseline exhibited limited separability; under conservative HDBSCAN settings, it may collapse into only a few clusters, corroborating its lower discriminative power on this dataset. These outcomes aligned with the qualitative findings in the 2D projections and montage panels, where deep backbones form better-separated manifolds and HDBSCAN isolates dense, clean micro-clusters that are well suited to batch labeling.

Although the overall clustering quality was similar between ResNet-50 and ViT-B/16, small but consistent differences emerged. ResNet-50 achieved slightly higher NMI (0.290) than ViT-B/16 (0.281). This effect was most pronounced on classes with sharp local boundaries (e.g., Short, Spur), where convolutional filters preserved edge continuity. By contrast, ViT-B/16 was more competitive on texture-like defects (e.g., Spurious Copper), consistent with the self-attention mechanism’s sensitivity to global patterns.

As shown in Table 4, bootstrap confidence intervals confirm that metric variations across encoders are modest and largely overlapping (ΔNMI ≤ 0.01). Only the ResNet-50 + k-means baseline was significantly lower (*p* = 0.041).

We also performed ablation studies on parameter choices. For bounding box expansion, cluster purity peaked at 0.624 with a 10 px margin, compared to 0.602 (0 px) and 0.613 (16 px) on ResNet50 with HDBSCAN. For HOG features, performance improved sharply up to 128D but plateaued at 256D. For ViT-B/16 embeddings, fusing layers L11 + L12 yielded the best NMI = 0.281, slightly outperforming the CLS token alone (0.275).

Regarding clustering algorithms, HDBSCAN with (min_cluster_size = 30, min_samples = 15) produced the most balanced trade-off between purity and coverage. Finally, CPU/GPU parity checks confirmed consistency, with negligible metric deviations (ΔNMI < 0.002; ΔARI < 0.002).

### 3.2. Macro- vs. Micro-Cluster Purity

From per-cluster label breakdowns, ResNet-50 + HDBSCAN achieved macro-purity ≈ 0.725 and micro-purity ≈ 0.624; ViT + HDBSCAN achieved macro-purity ≈ 0.743 and micro-purity ≈ 0.606. Macro-purity (unweighted) exceeding micro-purity (weighted) indicated many small but clean clusters, which was desirable for cluster-level batch labeling.

### 3.3. Qualitative Analyses

**Figure 3:**
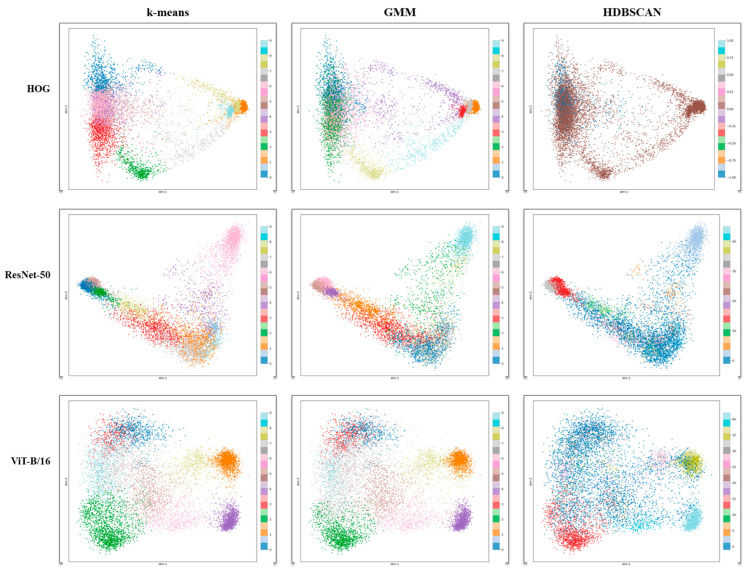
Two-dimensional projections (t-SNE) illustrating the visual separability of clusters.

Figure 3 shows two consistent patterns. First, deep encoders (ResNet-50, ViT-B/16) formed well-separated manifolds for dominant classes, while HOG exhibited diffuse or background-driven groupings, corroborating its lower quantitative agreement (Table 4). Second, method-specific structure was visible across columns. K-means yielded roughly spherical islands that reflected centroid partitions under K = 10; GMM revealed elongated or overlapping modes consistent with anisotropic covariance; and HDBSCAN isolated compact micro-clusters and a visible noise set, which explained its tendency to discover more clusters yet maintain high macro-purity. Also, regarding visual inspection of cluster-to-class alignment, Short and Open form compact, well-separated clusters, while Pollution overlaps substantially with Foreign Particles, which is consistent with the quantitative purity results.

Across encoders, ResNet-50 typically produced tighter, more compact islands than ViT-B/16 for the same clusterer, whereas ViT sometimes separated texture-frequency cues that benefit density-based discovery but could dilute ARI under fixed K partitions. These visual trends were consistent with the quantitative results in Table 5.

**Figure 4:**
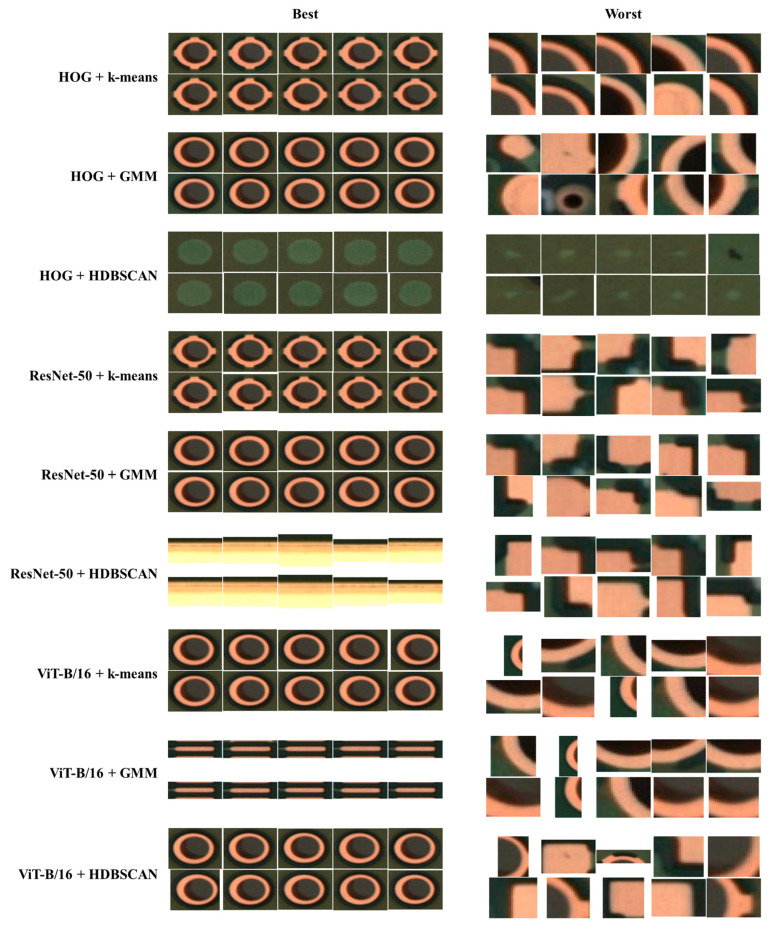
Best–worst cluster inspection across encoder–clustering algorithm pairs.

Figure 4 presents best–worst inspection across all encoder–clustering algorithm pairs, exposing method-specific failure modes that guide downstream prioritization and allocation.

Table 5 indicates that, across all nine encoder–clustering algorithm pairs, the best clusters were consistently near-pure (Best Purity ≈ 0.96–1.00) and typically captured canonically expressed majority classes (most often PSR Peel-Off; for HDBSCAN with deep encoders, tiny but perfectly pure Pollution clusters also appeared, e.g., sizes 14–35), whereas worst clusters were both larger and substantially mixed (Worst Purity ≈ 0.23–0.34; sizes frequently in the hundreds to thousands) and concentrated their ambiguity around PSR Peel-Off, Pollution, and, for some partitioning settings, Foreign Particles. Majority classes, such as PSR Peel-Off, reached higher purity (>0.80), while minority classes, such as Mouse-bite, dropped below 0.50 due to limited support and higher visual ambiguity. These quantitative differences provide context for overall macro-purity values and highlight the importance of imbalance awareness evaluation.

Beyond the quantitative metrics in Table 5, we further examined the clusters with the lowest purity to explore the underlying causes of failure. Representative “worst” clusters are illustrated in Figure 4, augmented with additional examples to capture the diversity of error modes. Three primary factors emerged as follows:

(i) Complex backgrounds. In some patches, high-frequency textures or soldering residues were visually dominant, causing defect signals, such as pinholes or scratches, to be absorbed into background patterns.
(ii) Lighting and contrast variations. Tiny or low-contrast defects (e.g., hairline scratches, faint pollution marks) were frequently misassigned due to uneven illumination or a low signal-to-noise ratio.
(iii) Overlapping class definitions. Semantic ambiguity between classes, such as Pollution vs. Foreign Particles or Spur vs. Spurious Copper, often led to mixed clusters, even under otherwise stable conditions.

These observations found that clustering errors were not random but systematically tied to image complexity and taxonomy overlap. Recognizing these root causes provided guidance for future refinements, such as adaptive pre-processing to normalize background intensity and revised labeling guidelines to reduce semantic overlap between defect categories.

### 3.4. Practical Labeling Impact

HDBSCAN surfaced dozens of compact, high-purity clusters; batches can be labeled (or rejected) with one decision each, minimizing per-image effort. K-means with K aligned to expected classes provides stable partitions at the dataset level, reducing rework when the class taxonomy is fixed. Across backbones, ResNet-50 achieved the best balance of separability and computational cost; ViT was close behind, and HOG was suitable mainly as a CPU-first baseline with dimensionality reduction. Collectively, these observations motivate a practical policy: employ HDBSCAN for initial batch acceptance and prioritization and then maintain stable partitions under a fixed taxonomy with k-means, defaulting to ResNet-50 embeddings unless resource or domain constraints dictate otherwise.

Beyond the reduction in annotation decisions, we quantified the time cost by assuming a realistic industrial annotation speed (≈3 s per image for manual labeling, plus 30–50% of that time for second-pass verification). Under this assumption, annotating 1000 images manually required ~50 min, whereas our clustering-based pipeline reduced the time to ~1.7 min, a ~30× reduction in wall-clock time. Scaling to 10,000 images, the cost gap widens to ~8.3 h vs. ~17 min. These estimates align with the observed decision reduction (~200×) but provide a more concrete measure in terms of absolute operator time.

### 3.5. Inter-Rater Agreement

Dual annotations were available for 2269 image patches. The overall inter-rater agreement, measured by Cohen’s κ, was 0.85, indicating substantial to almost perfect agreement according to Landis and Koch’s guideline [39]. This suggests that the annotation protocol was sufficiently consistent for practical use in semi-automatic labeling.

### 3.6. Generalization Across Imbalance Ratios

To test the robustness of our approach beyond the extreme imbalance of the original dataset (IR ≈ 1542), we conducted controlled down-sampling to create subsets with IR = 500 and IR = 1000. The results are summarized in Table 6.

Despite large changes in class priors, clustering performance remained stable; purity varied within 0.596–0.658, and NMI/ARI showed no systematic degradation (NMI = 0.222–0.250, ARI = 0.120–0.176). The number of clusters (K) also adjusted smoothly with data density (32 at IR = 500, 31 at IR = 1000, 46 at IR = 1542), confirming that HDBSCAN heuristic adapts to imbalance severity without fragmenting or collapsing minority classes. These findings support the idea that the proposed framework generalizes across different imbalance ratios.

## 4. Discussion

Industrial vision has pursued two main paths to reduce annotation burden. The first is anomaly detection, which models normality and flags deviations (e.g., deep one-class objectives, PaDiM, PatchCore, DRAEM) and has proven effective at localization without defect labels [7,8,9,10]. This line, however, typically stops at the ROI proposal stage, leaving open how to transform proposals into consistent class labels for supervised training downstream. The second path is semi- and weak supervision via embedding and cluster workflows. Features from pretrained encoders (Res-Net-50, ViT-B/16) are partitioned by k-means/GMM or density methods, such as HDB-SCAN, enabling annotators to label clusters rather than individual images [16,17,18,19,20,21]. Despite promising savings, prior art often remains method-centric, with limited treatment of operations constraints (auto-K selection, CPU-only fallbacks, memory safety on thousands of crops, and human-centered visualization) that are crucial in factory settings. In parallel, surveys across VI/PCB [1,2,3] and small-object detection [4,5,6] emphasize persistent obstacles—tiny, low-contrast targets against complex backgrounds and long-tailed class distributions—which systematically inflate labeling cost. Against this background, our work situates itself as a bridge between unsupervised localization and practical, scalable label production.

Figure 4 shows the best- and worst-purity clusters across encoder–clustering pairs. The best cluster exhibits near-pure membership, suggesting high intra-cluster cohesion for visually canonical instances. By contrast, the worst cluster mixes multiple categories, indicating ambiguous boundaries or taxonomy overlap (e.g., contamination versus surface specks). These observations motivate three concrete improvements: first, encoder refinement on hard negatives (contrastive fine-tuning) to enlarge inter-class margins; second, taxonomy grooming and micro-cluster consolidation to reduce label churn under rare subclasses; and third, verification tooling that prioritizes low-purity clusters for targeted human review (active selection), with merge/split actions logged for label propagation. Operationally, this best–worst analysis offers a pragmatic selection signal that focuses limited review capacity where it yields the largest purity gains.

The most immediate benefit was cluster-level batch labeling. A fully supervised, detector-first workflow required per-image decisions over thousands of samples; our approach reduced the decision count to the number of clusters. On this dataset, HDBSCAN discovered ~47 clusters with ResNet-50 and ~44 with ViT-B/16 (Table 4), implying an upper-bound decision compression factor of samples/clusters ≈ 200×.

In terms of operator time, assuming ~3 s per manual annotation, this reduction translates to ~50 min for 1000 samples versus ~1.7 min with our cluster-based pipeline—a ~30× wall-clock speed-up. Scaling to 10,000 images, the gap widens to ~8.3 h versus ~17 min. In practice, operators still reviewed boundary cases, but the image-to-group shift preserved most of the throughput gain. This matches evidence from semi-/weak-supervised pipelines that report substantial reductions in labeling cost when annotations are aggregated at the group level.

As summarized in Table 4 and Figure 3, ResNet-50/ViT + HDBSCAN yielded macro-purity > micro-purity (e.g., 0.725 vs. 0.624 for ResNet-50; 0.743 vs. 0.606 for ViT). This indicates many small, clean clusters, enabling one-shot acceptance/rejection of entire groups with limited precision loss. HDBSCAN’s explicit noise labeling [8] further concentrated human effort on ambiguous clusters and outliers (effective triage). When a fixed taxonomy was required, k-means with K = 10 attains the strongest ARI (e.g., 0.169 with ResNet-50; 0.158 with ViT), providing stable partitions at the dataset level that simplify longitudinal relabeling and change management. Operationally, this suggests a clear choice: HDBSCAN for high-mix, long-tailed lines, and k-means when class cardinality is fixed. These analyses provide mechanistic insight into the quantitative differences reported earlier. ResNet-50′s convolutional bias towards edges explains its advantage on boundary-centric classes, while ViT-B/16′s global receptive field aids texture-like classes. Class-wise purity analysis highlights that imbalance and visual ambiguity, rather than clustering algorithmic instability, account for the lower scores in minority classes. The enriched qualitative results—t-SNE with class labels and failure-case analysis—demonstrate that low-purity clusters are not random but arise from interpretable factors, such as background interference and taxonomy overlap. These findings support the validity of our method while clarifying its limitations and guiding future refinements.

While per-class κ could not be computed due to the absence of balanced dual annotations for all defect categories, the overall agreement (κ = 0.85) indicates that label noise is unlikely to compromise the validity of subsequent clustering and detection experiments. Future work will involve systematic per-class reliability assessment with additional raters.

PCB imagery is characterized by complex copper-trace backgrounds and tiny, low-contrast defects [1,2]. The proposed margin-aware cropping and deep embeddings (ResNet/ViT) preserve minimal local context while improving separability; the 2D projections (Figure 3) demonstrate island-like clusters for dominant classes and reduced background absorption compared to hand-crafted features. This directly addresses challenges emphasized in small-object-detection surveys—scale mismatch and low SNR—and reduces cognitive load during labeling by surfacing coherent, visually homogeneous groups.

From an operability perspective, the pipeline fits factory constraints in three respects.

- First, resource fit. HOG (+ incremental PCA) supports CPU-only scenarios; ResNet-50/ViT runs on a GPU or CPU.
- Second, scale and safety. The pipeline handles thousands of crops with memory-safe routines and exports artifacts for traceability, aiding audits and compliance.
- Third, human factors. Cluster montages enable at-a-glance verification of within-cluster consistency and borderline cases, boosting review speed without degrading label quality.

The workflow automatically reported Purity, NMI, AMI, and ARI, supplying quantitative evidence for cluster-level acceptance criteria and post hoc analysis of failure modes (e.g., over-/under-segmentation, background absorption). At the dataset level, we standardized imbalance diagnostics via the imbalance ratio (IR) and a Gini-style dispersion of class counts, facilitating comparisons across lines and lots and informing parameter schedules.

Operationally, we recommend three configurations suited to common factory scenarios.

- Bootstrapping at scale. ResNet-50 (or ViT) + HDBSCAN is used for surface high-purity micro-clusters for rapid initial labeling.
- Stable taxonomies/periodic re-builds. K-means with K = expected classes to maximize ARI and partition stability.
- Edge/CPU-only checks. HOG (+ incremental PCA) with HDBSCAN is a lightweight screening path (lower precision but operationally simple).

Taken together—normal-only training → ROI proposals (prior work) → cluster-based batch labeling (this work)—the pipeline targets the true industrial bottleneck, labeling latency and cost, rather than detector state of the art alone, while remaining traceable and auditable for deployment.

Several practical limitations and boundary conditions warrant consideration when interpreting these results and planning deployment. First, the dataset exhibits extreme imbalance (imbalance ratio ≈ 1542; Gini ≈ 0.642), which both motivates our approach and biases purity metrics toward majority classes. Second, although we tested multiple encoders and clusterers, the space of self-supervised encoders and contrastive fine-tuning remains under-explored here. Third, HDBSCAN’s behavior depends on scale parameters; we used data-driven heuristics, but broader robustness sweeps are warranted. Fourth, our analysis emphasizes cluster-level acceptance rather than pixel-accurate delineation. Extremely tiny or low-contrast defects (e.g., pinholes) may still require manual refinement or re-training at the detector stage. Finally, we did not directly compare downstream detector performance between cluster-labeled and fully manually labeled datasets. Thus, claims about alleviating industrial bottlenecks should be regarded as potential benefits rather than definitive evidence.

The proposed framework was validated on PCBs fabricated with a uniform FR-4 substrate and green solder mask, which are representative of mainstream production lines. However, material and coating variations—such as mask color (green, blue, black), copper thickness, or surface finish (matte vs. glossy)—can alter texture contrast and illumination response, thereby influencing embedding quality and cluster separability. Although we did not perform a cross-material validation in this study, the framework is designed to remain largely material-agnostic because its feature extraction relies on pretrained vision backbones (ResNet-50 and ViT-B/16) and clustering operates on normalized embeddings.

Mitigation strategies for observed failure modes include the following:

(i) Density retuning for HDBSCAN (e.g., co-scheduling minimum cluster size and minimum samples and using scale-normalized distances).
(ii) Class-conditional auto-K or evidence-based model selection for partitioners to avoid over-/under-partitioning.
(iii) Guided merge/split operations with cluster-level label propagation.
(iv) Targeted encoder refinement (lightweight contrastive fine-tuning with hard clusters and curriculum from coarse to fine).
(v) Data-centric normalization (illumination/background control, crop margin standardization) to reduce spurious boundaries.

For future work in preparation, we outline five directions.

(i) Encoder learning. Evaluate self-supervised or domain-adaptive encoders and metric-learning objectives to sharpen separability for rare/tiny defects, potentially improving both HDBSCAN purity and k-means ARI.
(ii) Interactive loops. Integrate active selection (hard clusters first), human feedback (merge/split logs), and label propagation to iteratively refine clusters and train a supervised detector in a curriculum from coarse to fine.
(iii) Taxonomy and imbalance handling. Explore cost-sensitive clustering objectives, auto-K with model evidence, and uncertainty-aware consolidation of micro-clusters to stabilize labels under shifting class definitions; continue reporting IR and Gini as standard imbalance diagnostics.
(iv) Material robustness. Extend evaluation across heterogeneous PCB materials (e.g., different solder mask colors and surface finishes) to quantify cross-material stability and investigate adaptive normalization or domain-adaptive encoder fine-tuning for improved generalization (see Appendix B).
(v) Downstream detection. Use cluster-confirmed labels to train tiny-object detectors and few-shot variants and explicitly compare detector accuracy trained on cluster-labeled versus manually labeled datasets. Closing this loop with pseudo-label repair could ultimately yield a continuous learning pipeline across product revisions.

## 5. Conclusions

This study presented a semi-automatic labeling framework for PCB defect inspection that integrates feature learning with density-based clustering and automatic granularity control. Our experiments demonstrate that the approach can substantially reduce annotation effort while maintaining clustering quality across different imbalance severities and defect types. By quantifying labeling-time savings and analyzing both class-wise purity and backbone-specific differences, we provide a more transparent assessment of the method’s practical value.

At the same time, several limitations must be acknowledged. First, we did not directly compare downstream detector performance between cluster-labeled and fully manually labeled datasets; therefore, claims regarding end-to-end industrial productivity gains should be interpreted as potential rather than definitive. Second, performance remains suboptimal for extremely tiny or low-contrast defects (e.g., pinholes), where cluster separability is inherently limited. Third, although we included modern self-supervised encoders as backbones, their comparative contributions remain under-explored and merit systematic evaluation in future work.

In conclusion, the proposed framework advances the state of semi-automatic labeling for PCB inspection by prioritizing annotation efficiency and adaptive cluster granularity. While not a complete solution to all industrial bottlenecks, it provides a foundation for reducing human labeling costs and opens avenues for integrating richer encoders and downstream detector validation in future research.

## Figures and Tables

**Table 1:** Class-wise counts and proportions.

Class	Count	Proportion (%)	Average Size (px)	Local Contrast	Labeling Difficulty
PSR Peel-Off	4625	49.44	79 ± 18	0.83	Low
Unknown	1337	14.29	—	—	Not applicable
Pollution	1302	13.92	36 ± 8	0.52	High
Foreign Particles	982	10.5	43 ± 10	0.59	Medium
PSR Skip	632	6.76	54 ± 11	0.66	Medium
Scratch	329	3.52	41 ± 9	0.63	Medium
Missing of Silk	57	0.61	28 ± 6	0.48	High
Spurious Copper	49	0.52	32 ± 7	0.51	High
Pin-Hole	38	0.41	24 ± 6	0.44	High
Mouse-bite	3	0.03	17 ± 5	0.4	High

Unknown = samples without a confirmed defect class (ambiguous or unlabeled). Average defect size and local contrast are computed from annotated regions; these metrics are unavailable for Unknown samples.

**Table 2:** Encoder configurations for embedding extraction.

Encoder	Resize	Embedding Dimension	Batch Size
HOG	224	26,244	-
ResNet-50	224	2048	64
ViT-B/16	224	768	64

**Table 3:** Clustering algorithms and main parameters.

Algorithm	Target Clusters	Distance Metric	Init/Seed
K-means	10	Euclidean	42
GMM	10	Gaussian likelihood	42
HDBSCAN	Variable	Euclidean	N/A

**Table 4:** Quantitative comparison across encoder–clusterer pairs.

Encoder	Clustering Algorithm	Purity (95% CI)	NMI (95% CI)	AMI (95% CI)	ARI (95% CI)	*p* vs. Best
HOG	K-means	0.535 [0.523–0.547]	0.149 [0.142–0.156]	0.147 [0.139–0.154]	0.060 [0.056–0.064]	<0.001 *
HOG	GMM	0.558 [0.545–0.571]	0.157 [0.149–0.165]	0.155 [0.147–0.163]	0.034 [0.031–0.037]	<0.001 *
HOG	HDBSCAN	0.501 [0.487–0.515]	0.022 [0.018–0.026]	0.021 [0.017–0.025]	0.044 [0.041–0.047]	<0.001 *
ResNet-50	K-means	0.617 [0.604–0.630]	0.255 [0.247–0.263]	0.253 [0.244–0.262]	0.169 [0.162–0.176]	0.043 *
ResNet-50	GMM	0.616 [0.603–0.629]	0.248 [0.240–0.256]	0.246 [0.237–0.255]	0.153 [0.146–0.160]	0.018 *
ResNet-50	HDBSCAN	0.624 [0.610–0.638]	0.290 [0.282–0.299]	0.283 [0.274–0.292]	0.178 [0.170–0.186]	—
ViT-B/16	K-means	0.604 [0.592–0.616]	0.227 [0.219–0.235]	0.225 [0.216–0.234]	0.158 [0.150–0.166]	0.271
ViT-B/16	GMM	0.599 [0.586–0.612]	0.232 [0.224–0.240]	0.230 [0.221–0.239]	0.129 [0.122–0.136]	0.042 *
ViT-B/16	HDBSCAN	0.606 [0.592–0.620]	0.281 [0.273–0.289]	0.274 [0.265–0.283]	0.174 [0.166–0.182]	0.27

“Best” = ResNet-50 + HDBSCAN; * *p* < 0.05 indicates a significant difference.

**Table 5:** Summary of best–worst inspection across encoder–clustering algorithm pairs.

Encoder	Clustering Algorithm	Best Cases			Worst Cases		
		Purity	Majority Class	Size	Purity	Majority Class	Size
HOG	k-means	0.96	PSR Peel-Off	929	0.27	PSR Peel-Off	763
HOG	GMM	0.98	PSR Peel-Off	906	0.28	Pollution	1631
HOG	HDBSCAN	1	PSR Peel-Off	20	0.34	Pollution	902
ResNet-50	k-means	0.97	PSR Peel-Off	1122	0.23	Foreign Particles	1015
ResNet-50	GMM	0.97	PSR Peel-Off	1187	0.26	Pollution	768
ResNet-50	HDBSCAN	1	Pollution	35	0.23	Pollution	3445
ViT-B/16	k-means	0.97	PSR Peel-Off	1226	0.28	PSR Peel-Off	887
ViT-B/16	GMM	0.97	PSR Peel-Off	1171	0.32	PSR Peel-Off	910
ViT-B/16	HDBSCAN	1	Pollution	14	0.26	PSR Peel-Off	4114

**Table 6:** Clustering performance under different imbalance ratios.

IR	Purity	NMI	AMI	ARI	Clusters
500	0.642	0.222	0.238	0.120	32
1000	0.596	0.250	0.301	0.176	31
1542	0.658	0.233	0.279	0.162	46

## Data Availability

Data are not publicly available due to proprietary manufacturing constraints but are available from the corresponding author upon reasonable request.

## Abbreviations

The following abbreviations are used in this manuscript:

VI	Visual Inspection
PCB	Printed Circuit Board
HOG	Histogram of Oriented Gradients
ViT	Vision Transformer
GMM	Gaussian Mixture Model
HDBSCAN	Hierarchical Density-Based Spatial Clustering of Applications with Noise
t-SNE	t-distributed Stochastic Neighbor Embedding
IR	Imbalance Ratio
NMI	Normalized Mutual Information
AMI	Adjusted Mutual Information
ARI	Adjusted Rand Index
BIC	Bayesian Information Criterion

## Appendix A

**Algorithm A1.** Semi-automatic labeling pipeline.
Input: Raw PCB images I 1: Crop defect ROIs → C = {c_1_,…,c_n_} 2: Extract embeddings E = fθ(C) (θ ∈ {HOG, ResNet-50, ViT-B/16}) 3: Dimensionality reduction (PCA → 128–256 D) 4: Cluster E using k-means, GMM, or HDBSCAN 5: Assign cluster IDs → cluster-level annotation 6: Optionally merge/split clusters via human feedback Output: Label set L for training detectors

## Appendix B. Practical Parameter Tuning Guide

Typical parameter ranges for adapting to different PCB materials and lighting.

– Crop margin: 8–12 px (larger for reflective surfaces).
– Contrast normalization (γ): 0.9–1.2, depending on illumination.
– HDBSCAN min_samples: 5–15 for low-contrast defects.

These heuristics can assist industrial engineers in quick deployment without retraining.
